# MicroRNA-183-5p protects human derived cell line SH-SY5Y cells from mepivacaine-induced injury

**DOI:** 10.1080/21655979.2021.1946358

**Published:** 2021-06-28

**Authors:** Qian Zhou, Ling Zhang

**Affiliations:** Department of Anesthesiology, Jingzhou Central Hospital, Jinzhou, Hubei, China

**Keywords:** Mepivacaine, miR-183-5p, PDCD4, apoptosis

## Abstract

With the gradual recognition of the side effects of local anesthetics, the nerve injury caused by local anesthetics has received growing attention. This research intended to delve into miR-183-5p changes in mepivacaine-mediated SH-SY5Y cell injury, as well as its modulatory mechanism on cell apoptosis. RT-qPCR was adopted for assaying miR-183-5p and PDCD4 mRNA expression. Our team respectively transfected miR-183-5p mimic and inhibitor to enhance or inhibit miR-183-5p function. We employed Western blot for detecting PDCD4 protein levels, as well as flow cytometry and Hoechst 33342/PI double staining for determining cell apoptosis rate. Additionally, our crew applied an ELISA kit for measuring TNF-α, IL-1β, IL-6, and IL-8 contents. The level of reactive oxygen species (ROS) production was examined by the Image-iT LIVE Green ROS detection Kit. As well as dual-luciferase reporter experiment for verifying the targeting link of miR-183-5p with PDCD4. In mepivacaine-induced cell apoptosis in SH-SY5Y cells, miR-183-5p expression was down-regulated. TNF-α, IL-1β, IL-6, and IL-8 contents were elevated. The rate of apoptosis increased visibly, cleaved caspase-3 and Bax levels waxed, whereas Bcl-2 level waned. MiR-183-5p could alleviate the damaging impact of mepivacaine. Dual-luciferase reporter experiments demonstrated that miR-183-5p directly targeted PDCD4. Collectively, we concluded that a high concentration of mepivacaine can cause SH-SY5Y cell damage, miR-183-5p functions crucially in mepivacaine-mediated cell damage. This study provides a theoretical basis for elucidating the mechanism of mepivacaine-induced nerve cell damage, and overexpressed miR-183-5p likely become a novel strategy to combat mepivacaine-induced nerve damage.

Abbreviations:

miRNA: Micro RNA; PDCD4: Programmed Cell Death 4; MDA: Malondialdehyde; SOD: Superoxide Dismutase; ROS: Reactive Oxygen Species; WT: Wild Type; Mut: Mutant; UTR: Untranslated Region; IL-6: Interleukin-6; IL-1β: Interleukin-1β; TNF-α: Tumor Necrosis Factor-α; IL-8: Interleukin-8; COX-2: Cyclooxygenase-2; iNOS: inducible NOS; MEP: Mepivacaine

## Graphical abstract

**Figure:**
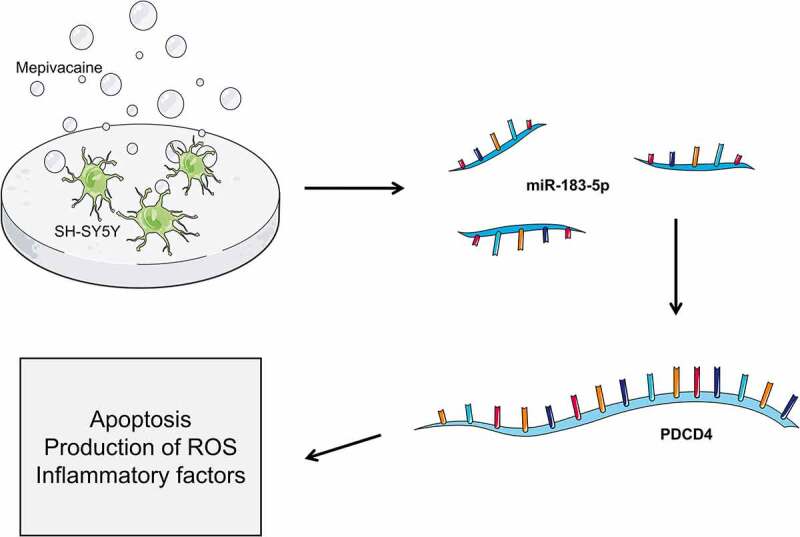

## 1. Introduction

Mepivacaine for injection, whose molecular formula is C_15_H_22_N_2_O·HCl, is an amide local anesthetic. Mepivacaine is stable, and when it reaches a certain concentration, it can attenuate the permeability of cations such as sodium and potassium to nerve cell membranes, thereby blocking the conduction of nerve impulses. It has a mild vasoconstriction effect, and its chemical structure, anesthetic effect, and toxicity are similar to lidocaine. Mepivacaine has been utilized clinically for many years, but the mechanism of nerve damage caused by it is still nebulous. Almost all local anesthetics can trigger neurotoxicity at high concentrations and large doses, and the injury has also been reported at clinical doses [1]. Transient neurologic syndrome (TNS) is the pain and paresthesia of the buttocks and lower limbs after the spinal anesthesia has completely recovered without special circumstances, and it is the primary manifestation of nerve injury with local anesthetics [2]. When mepivacaine is used for spinal and epidural anesthesia, the incidence of TNS is 6.4%, and it has been reported that it has a similar incidence of TNS to lidocaine [3,4]. Currently, the mechanism of mepivacaine-mediated cell injury is still equivocal, and the injury of mepivacaine must be fully paid attention to.

In recent years, the research on miRNA has become a momentous direction in the field of life sciences. MiRNA is a kind of endogenous non-coding single-stranded small RNA with 17–25 nt. It is produced from a 70–80 nucleotide length single-stranded RNA precursor (pri-RNAs) with a hairpin structure under the cutting of Dicer enzyme. It is extensively present in eukaryotes and is a set of short sequence RNA that does not code for protein. MiRNA has a high degree of conservation, timing, and tissue specificity among species. It binds to target mRNA through partial or complete complementarity, specifically induces the degradation or inhibits the translation of target gene mRNA, and participates in the adjustment of the biological signal pathway. MiRNAs are broadly distributed and governed by many target genes. MiRNAs are expressed in large quantities in the nervous system and function vitally in modulating neurogenesis, synaptic development, brain plasticity, etc., which are pivotal factors in modulating genes concerning dendritic morphology plasticity [5–7]. Thus, it is weighty to probe into the function and mechanism of miRNA. Additionally, it is known that miRNAs may mediate the inflammatory process, and anesthetics affect the resolution of inflammation [8]. Nonetheless, the influence of miR-183-5p on the nerve cell apoptosis induced by mepivacaine requires to be further investigation. It is not explicit whether interference with the function of miR-183-5p can counteract the nerve damage caused by mepivacaine.

In this work, mepivacaine was utilized for treating SH-SY5Y cells to make an *in vitro* nerve injury model. Then, RT-qPCR technology was adopted for detecting the changes in miR-183-5p expression in SH-SY5Y cells after mepivacaine-mediated injury. Subsequently, the changes in the levels of apoptosis proteins, inflammatory factors, and oxidative stress were detected. We further inquired into the modulatory influence of miR-183-5p on mepivacaine-mediated SH-SY5Y cell injury. Also, our findings could lay a new theoretical basis for developing sight in anesthesia injury.

## 2. Methods

### 2.1. Cell culture

Our team acquired human neuroblastoma cells SH-SY5Y from China Center for Type Culture Collection (CCTCC). We placed the SH-SY5Y cells in DMEM/F12 medium (Invitrogen, Carlsbad, CA, USA) supplemented with 10% FBS, as well as cultivated it in 5% CO_2_ incubator at 37°C. We added 10 mmol/L hydroxyethyl piperazine ethanesulfonic acid (HEPES) and penicillin and streptomycin 1.0 × 10^5^U/L each in the culture medium. When getting to approximately 80% confluence, we detached SH-SY5Y cells in the logarithmic stage making use of pancreatin, and passaged them. Mepivacaine at a concentration of 10 mmol/L was applied for treating SH-SY5Y cells, and Mepivacine-dissolved phosphate buffer was employed as a blank control. The induction time of mepivacaine was 24 h as previously described [9].

### 2.2. Cell transfection

Cell transfection was performed as described previously [10,11]. We seeded the SH-SY5Y cells treated with 10 mmol/L mepivacaine in a 6-well plate at a density of 5 × 10^5^/cm^2^ and placed them at 37°C, 5% CO_2_ incubator. 16 h later, cell transfection was performed. MiR-183-5p inhibitor, inhibitor-NC, miR-183-5p mimics, and mimics-NC were procured from Guangzhou RiboBio Co, Ltd. We used Lipofectamine ® 2000 (Invitrogen, Carlsbad, CA, USA) for transfecting SH-SY5Y cells according to the supplier’s instructions. RT-qPCR was exploited for assaying transfection efficiency.

### 2.3. Real-time quantitative PCR (RT-qPCR)

RT-qPCR was performed as described before [10]. Our crew extracted total RNA taking advantage of TRIzol reagent (Life Technologies Corporation) as well as RNeasy Mini kit (Qiagen, Duesseldorf, Germany), and then purified. For measuring and analyzing miR-183-5p, our team reverse-transcribed RNA exploiting TaqMan® MicroRNA Reverse Transcription Kit (Thermo Fisher Scientific). Besides, we used TaqMan® Universal Master Mix II (Applied Biosystems) for assisting TaqMan® MicroRNA analysis to carry out RT-qPCR. To determine and analyze PDCD4, we reverse-transcribed RNA samples applying Multiscribem reverse transcription kit (Applied Biosystems), and we completed RT-qPCR utilizing Fast START Universal SYBR Green Master (ROX) (Roche). We used the 2^−ΔΔCt^ method to analyze data. MiR-183-5p and PDCD4 mRNA expression were normalized to U6 and β-actin, respectively. The primer sequences were: miR-183-5p, 5ʹ-TATGGCACTGGTAGAATTCACT-3ʹ (Forward) and 5ʹ-ACGCTTCACGAATTTGCGT-3ʹ (Reverse); PDCD4, 5ʹ-AGAGACAGAAGAGCGGGGT-3ʹ (Forward) and 5ʹ-CCAGCATTTTCTGCAGGGTTT- 3ʹ (Reverse); U6, 5ʹ-TGCGGGTGCTCGCTTCGGCAGC-3ʹ (Forward) and 5ʹ-CCAGTGCAGGGTCCGAGGT-3ʹ (Reverse); β-actin, 5ʹ-CCTTCCTGGGCATGGAGTCCT-3ʹ (Forward) and 5ʹ-GGAGCAATGATCTTGATCTT- 3ʹ (Reverse).

### 2.4. Flow cytometric analysis of cell apoptosis

Cell apoptosis assay was performed as described previously [12]. Briefly, SH-SY5Y cells were exposed to with or without 10 mmol/L mepivacaine. After that, the cells were collected and washed three times with cold PBS, then resuspended in pre-cooled PBS at 4°C. Apoptotic cells were stained with dual-staining Annexin V-FITC-propidium iodide (PI) (catalog number 88–8006-72, Thermo Fisher Scientific, Waltham, MA, USA) and measured by FCM flow cytometer (BD Bioscience, San Jose, CA, USA).

### 2.5. Hoechst33342/PI double staining

Hoechst 33342 and propidium iodide are regarded as the markers of SH-SY5Y cells [13]. After treating SH-SY5Y cells with 10 mmol/L mepivacaine for 24 h, we discarded the original culture medium, rinsed the cells gently applying PBS solution, and added 0.5 mL Hoechst 33342 (catalog number 62,249, Thermo Fisher) and PI (catalog number R37108, Invitrogen) working solution at the same time. We placed it in an incubator under indoor temperature and fostered it for 15 min. After rinsing PBS, we observed and analyzed it utilizing a fluorescence microscope, counted the total number of SH-SY5Y cells and the number of apoptotic cells in the picture, and calculated the SH-SY5Y cell apoptosis rate.

### 2.6. Dual-luciferase reporter experiment

Luciferase reporter assay was performed as shown before [14]. Utilizing the StarBase database, our crew forecasted and attested that PDCD4 was likely the target gene of miR-183-5p. Our team inserted a wild-type PDCD4 3ʹ-UTR fragment comprising the miR-183-5p binding site into the luciferase reporter gene vector to establish a wild-type PDCD4-WT plasmid. Besides, we used gene mutation technology to mutate the binding site of miR-183-5p and PDCD4 3ʹ-UTR to build a mutant PDCD4-MUT plasmid. Meanwhile, we seeded HEK293 cells in the logarithmic growth phase at 5 × 10^4^ cells per well in a 24-well plate. When the cell growth confluence reached about 50%, our member utilized Lipofectamine® 2000 for transfection. We simultaneously transfected the PDCD4-WT and PDCD4-MUT plasmids with miR-183-5p mimics or mimics-NC, separately. Ultimately, we monitored the relative luciferase activity of cells according to the instructions of the luciferase activity detection kit.

### 2.7. Western blot analysis

Western blot assay was performed as described before [15]. Cells were subjected to Western blot under standards in a buffer comprising 50 mmol/L TRLs (pH 7.5), 150 mmol/L NaCl, 10% glycerol, 0.5% Nonidet P-40 as well as protease inhibitors (PMSF, Thermo Fisher). The antibodies used were anti-Cleaved caspase-3 (ab32042, Abcam, 1: 500), anti-Bcl-2 (ab32124, Abcam, 1: 1000), anti-Bax (ab32503, Abcam, 1: 1000), anti-PDCD4 (ab211501, Abcam, 1: 1000), anti-COX-2 (MA5-14568, Invitrogen, 1:1000), anti-iNOS (PA1-032, Invitrogen, 1:500) and anti-β-actin (ab8226, Abcam, 1: 2000). We used HRP-labeled anti-rabbit secondary antibody (ab9482, Abcam, 1: 1000) for incubating lasting 1 h. A chemiluminescence detection kit (ECL-plus kit or ECL-advance kit; Amersham) was employed for antibody detection, and ImageJ software was adopted for grayscale analysis.

### 2.8. Fluorimetric measurement of intracellular ROS

The Image-iT^TM^ LIVE Green Reactive Oxygen Species (ROS) Detection Kit obtained from Invitrogen (cat#136007, Invitrogen) was used to estimate ROS in live SH-SY5Y cells. This experiment was performed according to the manufacturer’s (Life technologies, D-339) recommended protocol. Cells were then seeded onto coverslips in 24-well plates 1 day before the experiment. The cells were then washed with HBSS, supplemented with 25 μM carboxy-H_2_DCFDA working solution, and incubated for 30 min at 37°C. Subsequently, the cells were washed again with HBSS, and the change in fluorescence was measured using a spectrofluorometer set at 485 nm excitation and 530 nm emission as described before [16].

### 2.9. Enzyme-linked immunosorbent assay (ELISA)

Enzyme-linked immunosorbent assay was performed as described previously [17,18]. Following the protocol of the kit (Jiancheng Bioengineering Institute, Nanjing, China), we determined reactive oxygen species (ROS) and malondialdehyde (MDA)(catalog number S0131S, Beyotime) contents and superoxide dismutase (SOD) (catalog number EIASODC) vitality. The cell suspension after treated with 5 mM mepivacaine for 24 h was collected and centrifugation was accomplished at 1500 r/min lasting 20 min to acquire cell supernatant. We collected the cell culture supernatant and assayed TNF-α, IL-6, IL-1β, and IL-8 contents in the cell culture based on the ELISA kit instructions.

### 2.10. Statistical analysis

Statistical analysis and data processing was accomplished by making use of SPSS 21.0 statistical software. We expressed the measurement data as mean ± SD. The inter-group differences in the measurement results were analyzed via one-way ANOVA. If the differences were statistically meaningful, the LSD test of pairwise comparison between groups was used. *P< 0.05.

## 3. Results

In this study, we conducted miR-183-5p mimics and inhibitor models in SH-SY5Y cells and performed the RT-qPCR, Western blot, FCM, dual-luciferase reporter as well as ROS detection to explore the function of mepivacaine on SH-SY5Y cell apoptosis. Moreover, we found that miR-183-5p targeted PDCD4 to exert a damaging effect in SH-SY5Y cell injury development.

### 3.1. Regulation of mepivacaine on miR-183-5p and PDCD4 expression in SH-SY5Y cells

**Figure 1.**
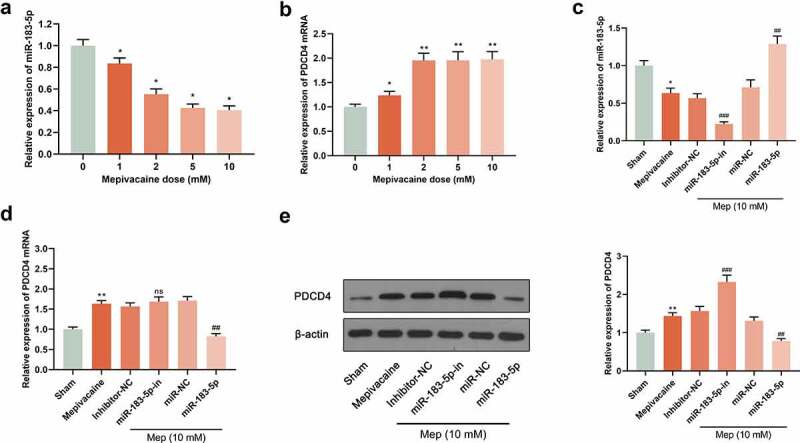
Effect of mepivacaine on miR-183-5p and PDCD4 expression in SH-SY5Y cells. (a) Mepivacaine could decline miR-183-5p level in a concentration-dependent manner. (b) Mepivacaine could augment PDCD4 mRNA expression level in a concentration-dependent manner. (c) We detected miR-183-5p expression in cells to verify the transfection efficiency. (d) PDCD4 mRNA level was measured after cell transfection and mepivacaine (10 mM) treatment. (e) PDCD4 protein level was measured after cell transfection and mepivacaine (10 mM) treatment.*P< 0.05, **P< 0.01 vs. sham group; #*P*< 0.05, ##*P*< 0.01, ###*P*< 0.001 vs. mepivacaine group

For digging into the impact of miR-183-5p on PDCD4 level, reverse transfection was applied for transfecting miR-183-5p mimics, miR-NC, miR-183-5p inhibitor, and inhibitor-NC into SH-SY5Y cells. 24 h later, we extracted total protein and RNA respectively. Subsequently, the influence of 0 mM, 1 mM, 2 mM, 5 mM and 10 mM mepivacaine on miR-183-5p and PDCD4 expression was delved into. The outcomes displayed that in contrast to the sham group, miR-183-5p expression of the mepivacaine group was observably decreased, whereas PDCD4 mRNA and protein expression noticeably increased (*P*< 0.05) (Figure 1(a,b)), implicating that miR-183-5p expression in SH-SY5Y cells increased after mepivacaine stimulation. We transiently transfected SH-SY5Y cell line to make it overexpressed or inhibited miR-183-5p and verified it in the cell (Figure 1(c)). Making use of Western blot and RT-qPCR, PDCD4 protein and mRNA expression was assayed. The outcomes disclosed that overexpressed miR-183-5p notably repressed PDCD4 mRNA and protein expression, and inhibition of miR-183-5p strikingly escalated PDCD4 mRNA and protein expression (Figure 1(d,e)).

### 3.2. MiR-183-5p targeted PDCD4

**Figure 2.**
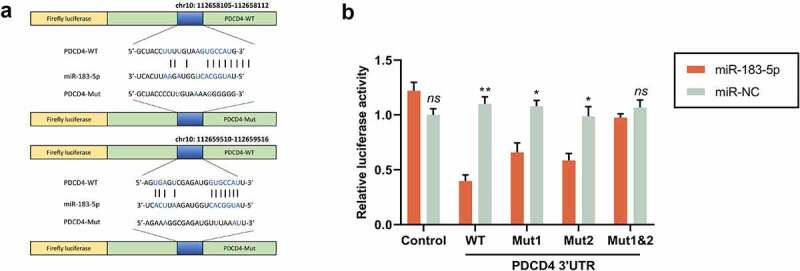
MiR-183-5p targeted PDCD4. (a) The forecasted target sequence of PDCD4 on the 3ʹ-UTR of miR-183-5p. Schematic diagrams showed the mutation in its binding site on the UTR. (b) Dual-luciferase reporter assay was conducted to verify the targeting relationship between miR-183-5p and PDCD4 in SH-SY5Y cells. Luciferase activity was decreased by miR-183-5p mimics. WT: wild-type; Mut: mutant. *P< 0.05, **P< 0.01 (* miR-183-5p vs. miR-NC)

For seeking the target genes modulated by miR-183-5p, we conducted a predictive analysis via the StarBase database. We evidenced that miR-183-5p has a binding site with PDCD4 3ʹUTR. Dual-luciferase activity detection evinced that, in comparison to the miR-NC group, the luciferase activity in WT-PDCD4 cells memorably dwindled, while the luciferase activity in Mut-PDCD4 cells did not change considerably in the miR-183-5p group. In comparison to the miR-NC group, the PDCD4 level in the miR-183-5p group saliently declined. In comparison with the inhibitor-NC group, PDCD4 expression in the miR-183-5p inhibitor group was sensibly increased (*P*< 0.05) (Figure 2(a,b)). The above consequences implicated that miR-183-5p could target and modulate PDCD4.

### 3.3. Regulation of miR-183-5p on mepivacaine-induced apoptosis

**Figure 3.**
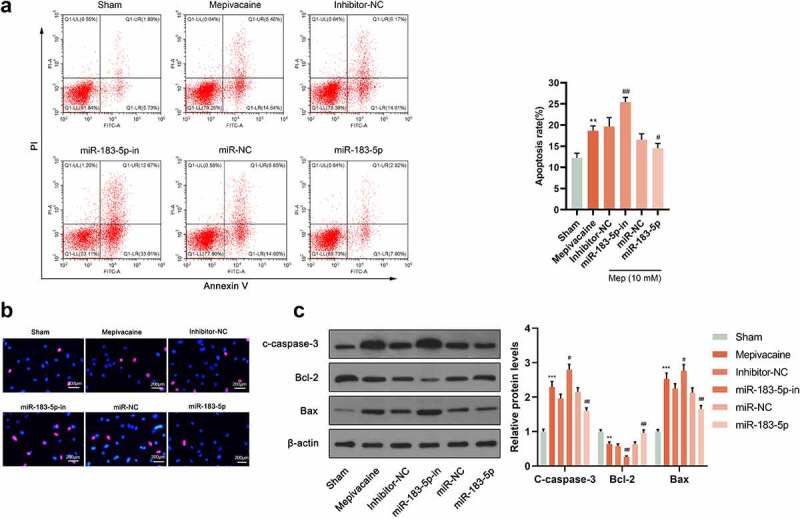
Influence of miR-183-5p on apoptosis induced by mepivacaine. (a) Apoptosis was quantified by flow cytometry using propidium iodide exclusion. (b) Hoechst33342/PI double staining to monitor the apoptosis rate. (c) Western blot to further determine C-caspase-3, Bax, and Bcl-2 expression levels. MiR-183-5p mimics and miR-183-5p inhibitor changed the expression level of these proteins.*P< 0.05, **P< 0.01 vs. sham group; #*P*< 0.05, ##*P*< 0.01 vs. mepivacaine group

For looking into miR-183-5p function in mepivacaine-induced nerve damage, we used mepivacaine to act on SH-SY5Y cells. Meanwhile, we successfully constructed a cell line transfected with miR-183-5p inhibitor or mimic. Flow cytometry analysis and Hoechst33342/PI double staining manifested that when 10 mmol/L mepivacaine was added to the culture medium, considerable red fluorescent apoptotic and dead cells were visible in the visual field. Overexpressed miR-183-5p saliently repressed mepivacaine-induced SH-SY5Y cell apoptosis rate. Furthermore, inhibiting miR-183-5p could brilliantly promote mepivacaine-induced SH-SY5Y cell apoptosis rate (Figure 3(a,b)). In comparison to the Sham group, cleaved caspase-3 and Bax protein levels in SH-SY5Y cells of the mepivacaine group were blatantly waxed, and Bcl-2 protein expression manifestly waned. Overexpressed miR-183-5p could inhibit the cleaved caspase-3 and Bax expression caused by mepivacaine while increasing Bcl-2 expression. On the other hand, inhibiting miR-183-5p exerted the opposite effect (Figure 3(c)).

### 3.4. miR-183-5p modulated oxidative stress level

**Figure 4.**
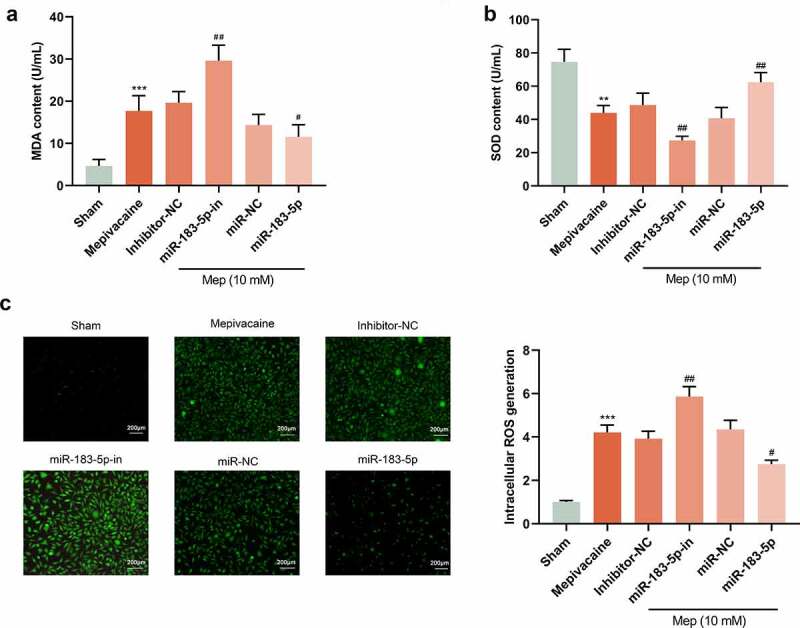
MiR-183-5p attenuated ROS and oxidative damage in SH-SY5Y cells. (a~b) ELISA kit to assay the content of malondialdehyde (MDA) and superoxide dismutase(SOD). (c) Qualitative detection of the steady-state levels of intracellular reactive oxygen species (ROS) by fluorescence microscopy.**P< 0.01, ***P< 0.001 vs. sham group; #*P*< 0.05, ##*P*< 0.01 vs. mepivacaine group

For better comprehending miR-183-5p function in mepivacaine-induced oxidative stress damage in SH-SY5Y cells, we also transfected SH-SY5Y cells with miR-183-5p inhibitor, as well as successfully diminished miR-183-5p expression. The content of oxidative stress-related factors in the cells was further tested. Compared with the Sham group, mepivacaine treatment patently raised MDA contents in SH-SY5Y cells, while lessening the activity of SOD (Figure 4(a,b)). To investigate whether miR-183-5p plays a role in the regulation of ROS production, we measured intracellular ROS production. Mepivacaine treatment resulted in increased ROS production. Furthermore, miR-183-5p was able to limit mepivacaine-induced ROS generation and inhibition of miR-183-5p treatment-induced excessive ROS generation (Figure 4(c)). About oxidative stress, it is caused by an imbalance between ROS production and ROS clearance pathways [19]. Overexpressed miR-183-5p could reverse the influence of mepivacaine on cellular oxidative stress factors while inhibiting miR-183-5p harbored the opposite impact. These outcomes indicated that miR-183-5p could lessen oxidative stress levels and abate cell damage.

### 3.5. Impact of miR-183-5p on the release of inflammatory factors from SH-SY5Y cells

**Figure 5.**
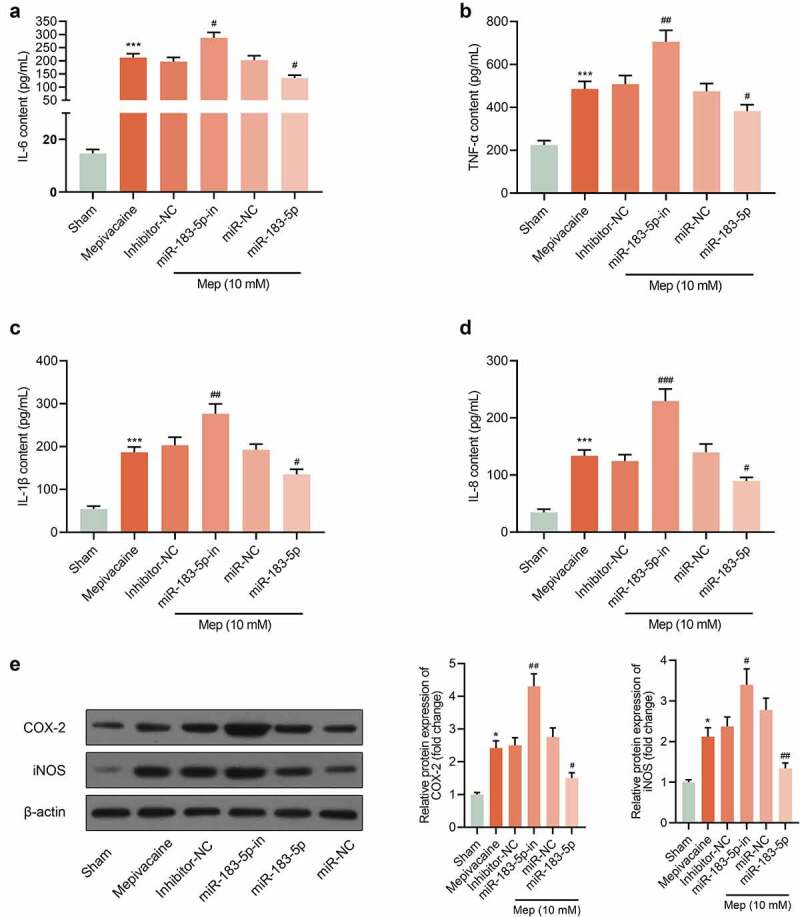
Impact of miR-183-5p on the release of inflammatory factors from SH-SY5Y cells. (a~d) Impact of miR-183-5p on the secretion of IL-6, TNF-α, IL-1β, and IL-8 by SH-SY5Y cells. (e) The protein expression of COX-2 and iNOS in the groups. β-actin was used as an invariant control for calculating protein fold changes. ***P< 0.001 vs. Sham group; #*P*< 0.05, ##*P*< 0.01, ###*P*< 0.001 vs. mepivacaine group

Compared with the Sham group, IL-6, TNF-α, IL-1β and IL-8 contents of SH-SY5Y cells in the mepivacaine group prominently declined. In contrast to the Mepivacaine+Inhibitor-NC group, the contents of the above-mentioned inflammatory factors in the mepivacaine+miR-183-5p inhibitor group eminently expanded. Overexpressed miR-183-5p held the opposite influence (Figure 5(a–d)). In addition, the expression of COX-2 and iNOS was increased after mepivacaine treatment, while miR-183-5p decreased the expression of COX-2 and iNOS (Figure 5(e)). Those implied that miR-183-5p could repress the release of cellular inflammatory factors induced by mepivacaine stimulation.

## 4. Discussion

Anesthetics are extensively applied in modern medicine, making safety issues a major health issue. Although the incidence of neurotoxicity induced by anesthetics is low, the prevention methods for these events are not adequately known. Hereby, we concentrated on the association of miR-183-5p with mepivacaine-mediated nerve damage.

MiRNAs function significantly in modulating neurotoxicity caused by anesthetics. For instance, miR-153 protects against isoflurane-induced neurotoxicity through Nrf2/ARE cells [20]. MiR-132 plays an important in the modulation of bupivacaine-induced neurotoxicity through IGF1R [21]. miR-497-5p down-regulates BDNF expression, which is recognized to be a defensive factor for nerve damage and TrkB/PI3K/Akt pathway activation, thereby aggravating the narcotic nerve damage caused by ketamine [22]. Nonetheless, few studies have inquired into the probable mechanism of miR-183-5p modulating mepivacaine-induced cellular neurotoxicity. In previous studies of nerve cells, miR-183-5p was confirmed to be involved in neuronal stress and dopamine secretion activities [23,24].In the current research, our consequences unveiled that in SH-SY5Y cells, miR-183-5p expression was preeminently reduced after mepivacaine treatment, while overexpressed miR-183-5p could outstandingly reverse this impact. Furthermore, overexpressed miR-183-5p could substantially attenuate mepivacaine-induced cell growth inhibition and apoptosis. In summary, mepivacaine induced the toxicity of SH-SY5Y cells via down-regulating miR-183-5p expression.

Owing to the consumption of oxygen is not proportional to its weight, and the lack of related enzymes, the nervous system is more fragile to the aggression from free radicals, especially hippocampal neurons. Hippocampal neurons are more assailable from free radicals than other neurons for the reason that they contain a lot of unsaturated fatty acids [25,26]. In addition to directly causing oxidative damage, oxidative stress can also oxidize protein sulfhydryl groups, thereby repressing the cellular uptake of glutamate. Glutamate is a monumental amino acid neurotransmitter in the central nervous system. Abnormal concentrations of glutamate can cause excitotoxicity. Moreover, oxidative stress can also reduce the potential of the mitochondrial membrane, leading to DNA damage, lipid and protein structure changes, and ultimately cell apoptosis [27]. Apoptosis is a process of programmed cell death that is regulated by genes. Its morphological changes are dominantly manifested in the condensation of nuclei, chromatin agglutination, and finally the formation of apoptotic bodies [28]. PDCD4 is capable of regulating axon growth by selectively controlling the translation process, suppressing axon growth-associated genes, and is adjusted in response to neural damage [29]. In retinal ischemia-reperfusion, lncRNA H19/miR-21/PDCD4 axis can directly adjust microglia relaxation and neuron damage caused by I/R [30]. Also, PDCD4 is governed by miR-340-5p, which can alleviate the nerve damage induced by hypoxia-glucose deprivation/reoxygenation through activating the PI3K/AKT signal pathway of rat hippocampal neurons [31]. Besides, in Alzheimer’s disease, miR-212 modulates PDCD4 through PI3K/AKT signaling pathway to allay Aβ25-35-induced neurotoxicity [32].

Also, some limitations existed in this work, one of which is the lack of research on factors such as surgical simulation. The influence of surgical stress, pain, inflammation, and tissue trauma on nerve injury is not thoroughly understood. Animal models are evincing that untreated pain can also give rise to nerve cell apoptosis and increased neurodegeneration [33]. On account of scanty particular animal models of mepivacaine-mediated neurotoxicity damage, current studies could not confirm the direct causal relationship between changes in miRNA expression and mepivacaine injury. We used a single miRNA, miR-183-5p, for verification, and the results probably had the risk of false-positive rates. Therefore, we need further research to figure out the function of differentially expressed miRNAs in mepivacaine-mediated SH-SY5Y cell damage.

All in all, our work has illustrated the causative role of miR-183-5p targeting PDCD4 to modulate the mepivacaine-mediated neurotoxicity. This discovery is conducive to digging into a new clinical therapy for neural injury in anesthesia. Nonetheless, when the mepivacaine-induced regulation of miR-183-5p happens in vivo, especially in humans, it is indistinct whether the *in vitro* results we obtained are true. Therefore, in subsequent research, we will focus on *in vivo* studies, which will undoubtedly widen our knowledge of the neurotoxicity triggered by mepivacaine.

## 5. Conclusion

Taken together, it is the first time for our study to provide a new finding that mepivacaine suppressed miR-183-5p expression, and miR-183-5p suppressed the apoptosis of SH-SY5Y cells *in vitro*, the up-regulation of miR-183-5p can be used for treated nerve injury caused by mepivacaine in pathological settings.

## Data Availability

The data used to support the findings in this study are available from the corresponding author upon request.

## Research highlights

- miR-183-5p was decreased in mepivacaine-treated SH-SY5Y cells.
- miR-183-5p repressed SH-SY5Y cell apoptosis.
- miR-183-5p suppressed the production of ROS and inflammatory factors.
- miR-183-5p targeted PDCD4.
